# Atypical microbial infections of digestive tract may contribute to diarrhea in mucopolysaccharidosis patients: a MPS I case study

**DOI:** 10.1186/1471-2431-5-9

**Published:** 2005-05-09

**Authors:** Grzegorz Węgrzyn, Julianna Kurlenda, Anna Liberek, Anna Tylki-Szymańska, Barbara Czartoryska, Ewa Piotrowska, Joanna Jakóbkiewicz-Banecka, Alicja Węgrzyn

**Affiliations:** 1 Department of Molecular Biology, University of Gdańsk, Kładki 24, 80-822 Gdańsk, Poland; 2 Department of Bacteriology, Provincial Hospital, Nowe Ogrody 1-6, 80-803 Gdańsk, Poland; 3 Department of Pediatrics, Children's Gastroenterology and Oncology, Medical University of Gdańsk, Nowe Ogrody 1-6, 80-803 Gdańsk, Poland; 4 Department of Metabolic Diseases, The Children's Memorial Health Institute, Al. Dzieci Polskich 20, 04-736 Warsaw, Poland; 5 Department of Genetics, Institute of Psychiatry and Neurology, Sobieskiego 9, 01-957 Warsaw, Poland; 6 Department of Genetics and Marine Biotechnology, Institute of Oceanology, Polish Academy of Sciences, Św. Wojciecha 5, 81-378 Gdynia, Poland; 7 Laboratory of Molecular Biology (affiliated with the University of Gdańsk), Institute of Biochemistry and Biophysics, Polish Academy of Sciences, Kładki 24, 80-822 Gdańsk, Poland

## Abstract

**Background:**

Mucopolysaccharidoses are heritable, metabolic diseases caused by deficiency in an activity of one of specific lysosomal enzymes involved in degradation of mucoplysaccharides (glycosaminoglycans). Among many medical problems of patients with mucopolysaccharidoses, there are frequent episodes of diarrhea of unknown etiology.

**Case presentation:**

A girl, diagnosed enzymatically for mucopolysaccharidosis type I (deficiency of α-L-iduronidase) at the age of 3 years and 9 months, was investigated until the age of 5 years and 4 months. Frequent loose stools and episodes of diarrhea, often accompanied by vomiting, were encountered. Detailed microbiological analyses were performed and atypical microbial infections (most often enetropathogenic *Escherichia coli*, but also other species, like *Pseudomonas aeruginosa *or *Staphylococcus aureus*, as well as adenoviruses) of the digestive tract were found in most severe diarrhea episodes. Often, isolations of pathogenic bacterial strains from stools of the investigated patient suffering from diarrhea were not obvious during the first screening, and only detailed microbiological studies, including re-isolation of colonies, gave the results of isolation of particular pathogenic strains (especially in the case of enetropathogenic *E. coli*).

**Conclusion:**

We conclude that atypical microbial infections of digestive tract may contribute significantly to diarrhea in mucopolysaccaridosis patients. Since isolated strains were not typical and their isolation was often possible only after detailed investigation (not during a standard screening), such atypical microbial infections of digestive tract of mucopolysaccharidosis patients could be usually overlooked to date. Importantly, these atypical infections could be effectively treated with antimicrobial agents.

## Background

Mucopolysaccharidoses (MPS) are rare genetic diseases, inherited in an autosomal recessive manner (except MPS type II, which is X-linked) [1]. Each MPS is caused by deficiency in an activity of one of specific lysosomal enzymes involved in degradation of mucoplysaccharides (glycosaminoglycans, GAGs). When activity of such an enzyme is impaired, GAGs are not destroyed but their accumulate in lysosomes of virtually every cell of the body as well as outside of cells [1,2]. Depending on the kind of a deficient enzyme, several types of mucopolysaccaridosis can be distinguished [3].

Cells of MPS patients do not perform properly, which leads to progressive damage throughout the body (mainly connective tissue), including the heart, respiratory system, bones, joints and, in some cases, central nervous system [1,3]. In most cases the disease is fatal, however, prediction of its severity and clinical progress is usually difficult, even when biochemical and genetic data are available [4]. Among many medical problems, MPS patients suffer from frequent episodes of loose stools and diarrhea. Although mechanisms of other symptoms of MPS are known to some extend, the cause of diarrhea remains obscure [1,3]. One might speculate that it could be a defect in the autonomic nervous system caused by storage of glycosaminoglycans, and resultant abnormal motility of the bowel would cause the diarrhea. However, few evidences can support such a hypothesis. Therefore, we have investigated factor(s) that might contribute to this problem of MPS patients. Here, a case report is presented, in which a MPS I patient was investigated, however, it is worth mentioning that diarrhea is also a common problem for MPS patients of other types. MPS I is caused by a defect in the gene coding for α-L-iduronidase [5]. We found that episodes of diarrhea were accompanied by atypical microbial infections that could be treated by antimicrobial agents. Recently, a specific treatment of MPS I became available, since recombinant human α-L-iduronidase (Aldurazyme^®^, laronidase) has been shown to be effective [6,7]. During three months of treatment of the patient with Aldurazyme, no diarrhea episodes occurred, strongly suggesting that previous episodes results indirectly from MPS rather than from putative, MPS-independent, enhanced susceptibility of the patient to microbial infections of intestine.

## Case presentation

A girl, diagnosed enzymatically for MPS I (McKusick's OMIM (MPS I, Hurler syndrome, Scheie syndrome): 25280) at the age of 3 years and 9 months, was investigated until the age of 5 years and 4 months. Genetic analysis (performed by us) indicated that one allele of the α-L-iduronidase gene contains a mutation causing a common Q70X truncation in the enzyme amino acid sequence. Further studies (performed at Consorzio Genetica Moleculare Umana, Monza, Italy, supervised by Prof. Andrea Biondi and coordinated by Genzyme Co.) confirmed the presence of this allele and indicated that the second mutation is an in frame deletion of three base pairs (codon 349), resulting in the loss of aspartic acid residue (Δ349). The investigated MPS I patient suffered, between other symptoms characteristic for this disease, from frequent diarrhea or loose stools, often accompanied by vomiting. No obvious contacts with persons suffering from intestinal infections, including family members (who also had no documented chronic or often diarrhea), were noticed shortly before or during these episodes. During the most severe episodes of diarrhea, detailed microbiological analyses of stools were performed. The patient was never treated with antibiotics before stool collection, thus, results of microbiological assays were not affected by such a treatment. Bacterial, viral or fungal infections were detected in all these analyses (Table 1).

**Table 1:** History of the most severe diarrhea events caused by microbial infections of intestine of the MPS I patient

Age of the patient^a^	Detected pathogenic microbial strain(s)	Antimicrobial agent(s) used	Special remarks
11 months	*E. coli *(group C)	Trimethoprim and sulphamethoxazole	Severe diarrhea and vomiting, metabolic acidosis, hospitalization and i.v. rehydration required
21 months (1 y. 9 mo.)	*E. coli *(group C)	Cefuroxime	Initially applied trimethoprim and sulphamethoxazole did not give a therapeutic effect
27 months (2 y. 3 mo.)	*E. coli *(group A)	Nifuroxazide	None
28 months (2 y. 4 mo.)	*P. aeruginosa*	None	Tablets containing *Lactobacillus rhamnosus *were used to normalize intestinal flora
29 months (2 y. 5 mo.)	*E. coli *(group C)	Furazidin	None
45 months (3 y. 9 mo.)	Adenoviruses, *C. albicans *(in addition, *H. parainfluenzae, S. viridans *and *Neiserria *sp. were isolated from oral cavity)	Cefuroxime, fluconazole, trimethoprim and sulphamethoxazole	Very severe diarrhea and vomiting, severe dehydration and electrolyte depletion, metabolic acidosis, severe stomatitis. Hospitalization and parenteral nutrition required
49 months (4 y. 1 mo.)	Adenoviruses	None	None
50 months (4 y. 2 mo.)	*E. coli *(group C)	Furazidin, trimethoprim and sulphamethoxazole	Severe diarrhea, hospitalization and i.v. rehydration required
54 months (4 y. 6 mo.)	Adenoviruses	None	None
55 months (4 y. 7 mo.)	*E. coli *(group C)	Furazidin	None
56 months (4 y. 8 mo.)	*E. coli *(group C), *S. aureus*, *C. albicans*	Furazidin, fluconazole	None

^a ^Age of the patient is provided in months as well as in years (y.) and months (mo.) in parentheses

Isolation and preliminary analyses for identification and characterization of bacteria, viruses and fungi were performed using S-F selenium, blood agar, CNA, McConkey, SS, XLD, Sabouraud, Kligler and Singer, and BHI media (purchased from Becton Dickinson, Biomed or Bimex). Detailed analyses were performed using biochemical assay kits ID GN 32 (BioMerieux), EN-COCCUStest (Lachema) and ATB ID 32 C (BioMerieux), and immunological tests (kits purchased from Immunomed and BioMarieux). The microbiological assays were performed according to the semi-quantitative method, described previously [8]. Non-enteropathogenic strains of *Escherichia coli*, strains of *Enterococcus faecalis *and small amounts of other bacteria belonging to Enterobacteriaceae were considered as normal aerobic bacterial flora. Identification of enteropathogenic *E. coli *strains was performed using a specific latex-immunological assay (purchased from Biomex). In this assay, 10 randomly chosen *E. coli *colonies (classified on the basis of microbiological and biochemical tests described above) were streak onto nutrient agar plates, and following 18 h incubation at 35°C bacteria were tested immunologically using the Biomex kit. The O antigens (characteristic for enteropathogenic *E. coli *strains but not for other strains of this bacterium), were detectable in this assay, and corresponding strains, bearing particular antigens, were divided into following groups: group A (antigens O26, O55, O111, O127 and O142), group B (antigens O86, O119, O124, O125, O126 and O128) and group C (antigens O18, O25, O44 and O114).

As summarized in Table 1, all viral infections were caused by adenoviruses, and tests were negative after diarrhea was finished. Most bacterial infections were atypical. Enteropathogenic *Escherichia coli *belonging to group A or, more frequently, to group C, was detected in over 50% of positive tests. These bacteria may cause diarrhea in infants, but they are very rare agents causing intestinal problems in older children and adults, thus the tests for detection of these strains are usually not performed in routine studies if the patient is not an infant [9,10]. *Pseudomonas aeruginosa, Staphylococcus aureus *and *Candida albicans *were also detected, though significantly less frequently than *E. coli *group C. These species can be detected also in stools of healthy persons. Thus, in our assays, they were considered potential etiological agents only when they dominated quantitatively over other microbes treated as physiological flora, and the dominance was significant. In fact, during the episode at the age of 28 months (Table 1), *P. aeruginosa *was the only bacterium isolated from the stool sample. Although no antibiotic therapy was applied in this case (due to problems with efficacy of treatment of *P. aeruginosa *infections with antibiotics), the symptoms of diarrhea disappeared and normalization of the intestinal flora was achieved after using tablets containing *Lactobacillus rhamnosus*. This strongly suggests a significant contribution of the *P. aeruginosa *infection to diarrhea. At the age of 45 months, when the patient suffered from a very severe diarrhea and vomiting (Table 1), apart from adenoviruses, *C. albicans *was detected in large quantities in stool samples, and large amounts of *Haemophilus parainfluenzae, Streptococcus viridans *and *Neiserria *sp. were isolated from oral cavity. Although *S. viridans *and *Neiserria *are considered as a part of normal bacterial flora of oral cavity in infants, their abundance, together with that of *H. parainfluenzae *was accompanied with significant depletion of the rest of physiological flora. During the episode of diarrhea at the age of 56 months (Table 1), apart from isolation of enteropathogenic *E. coli*, severe depletion of physiological flora and presence of large quantities of *S. aureus *and *C. albicans *was found. The enteropathogenic *E. coli *strain might be the major bacterial contributor to this infection as treatment with furazidin (which is generally not used for treatment of *S. aureus *infections) and fluconazole (an antifungal agent) was effective.

It is worth mentioning that isolations of pathogenic bacterial strains from stools of the investigated patient suffering from diarrhea were often not obvious during the first screening, especially in the case of enteropathogenic *E. coli*. Only detailed microbiological studies, including re-isolation of colonies, gave the results of isolation of particular pathogenic strains (summarized in Table 1). We suppose that this could be the explanation of the fact that atypical microbial infections of digestive tract of MPS patients were usually overlooked to date. On the other hand, these infections apparently caused diarrhea. In most episodes accompanied by isolation of bacterial infection, drug sensitivities were determined and antibiotics were used accordingly (Table 1). After this treatment, diarrhea was resolved and pathogenic bacteria were not detected in stools. Similar results were obtained after therapy with an antifungal drug, when *C. albicans *was detected (data not shown).

Intestinal flora was normal when tested in periods of normal stools (microbiological tests were performed 5 times during such periods as control experiments; data not shown). This provides an additional support for the hypothesis that isolated pathogenic microbial strains caused diarrhea, rather than the patient was a carrier of an atypical bacterial flora.

The patient was treated with Aldurazyme (laronidase; recombinant human α-L-iduronidase), by weekly infusions of 100 U per 1 kg of body weight, during last three months of the study, and no episodes of diarrhea occurred during that period. This indicates that episodes of severe diarrhea, listed in Table 1, resulted directly or indirectly from accumulation of GAGs, rather than from a putative, unrelated to MPS I, enhanced susceptibility of the patient to microbial infections of intestine.

## Conclusion

On the basis of the results presented in this report, we suggest that microbial infections of intestine may contribute significantly to episodes of diarrhea in MPS patients. Is it clear that influence of other factors and agents, for example those resulting from dietary incompliance, cannot be excluded. Nevertheless, it seems clear that the most severe diarrhea events in the MPS I patient were caused by microbial infections, as when pathogenic bacteria or fungi were isolated, antibiotic treatment always resulted in normalization of stools. One may speculate that GAGs, which accumulate in MPS patients' digestive tract, could provide a favorable medium for microbial growth. Under such conditions, any disturbance in the content of intestinal flora would make an overgrowth of a pathogenic strain significantly more likely than in a healthy person. Finally, accumulated GAGs might also impair production and/or action of IgA, a class of antibodies predominating in an intestine [11,12], which would make a patient more sensitive to microbial infections. One might consider that in MPS patients, diarrhea arises directly from GAG accumulation and partial dysfunction of autonomous nervous system. Although we cannot exclude such a possibility, the arguments listed above indicate that atypical microbial infections of digestive tract may contribute significantly to diarrhea in these patients.

## Abbreviations

MPS, mucopolysaccharidosis; GAG, glycosaminoglycan. y., years; mo., months

## Competing interests

The author(s) declare that they have no competing interests.

## Authors' contributions

GW was the main coordinator of this study and drafted the manuscript. JK performed all microbiological analyses. AL and AT-S participated in the design of the study and performed clinical observations. BC performed biochemical analyses and participated in coordination of the study. EP and JJ-B performed genetic analysis. AW participated in the design of the study and contributed to preparation of the manuscript draft. All authors read and approved the final manuscript.

## Pre-publication history

The pre-publication history for this paper can be accessed here:


